# Nrf2 Activated by PD-MSCs Attenuates Oxidative Stress in a Hydrogen Peroxide-Injured Retinal Pigment Epithelial Cell Line

**DOI:** 10.3390/antiox14111279

**Published:** 2025-10-25

**Authors:** Se Jin Hong, Dae-Hyun Lee, Jeong Woo Choi, Hankyu Lee, Youngje Sung, Gi Jin Kim

**Affiliations:** 1 Division of Life Sciences, Department of Life Science, Graduate School, CHA University, Seongnam-si 13488, Republic of Korea; sejin1451@gmail.com (S.J.H.); ldh92426@gmail.com (D.-H.L.); 2 Department of Biomedical Science, CHA University, Pocheon-si 11160, Republic of Korea; why12525@chauniv.ac.kr (J.W.C.); hglee@plabiologics.com (H.L.); 3 PLABiologics Co., Ltd., Seongnam-si 13522, Republic of Korea; 4 Department of Ophthalmology CHA Bundang Medical Center, CHA University, Seongnam-si 13488, Republic of Korea; noblelion@chamc.co.kr

**Keywords:** age-related macular degeneration, oxidative stress, KEAP1, Nrf2, mitochondrial dynamics, placenta-derived mesenchymal stem cells, ARPE-19

## Abstract

Age-related macular degeneration (AMD) is a retinal degenerative disease caused by oxidative stress. Thus, we aimed to reduce oxidative stress through the use of placenta-derived mesenchymal stem cells (PD-MSCs). To induce oxidative stress in ARPE-19 cells, we treated them with 200 µM hydrogen peroxide (H_2_O_2_) for 2 h and then cocultured them with PD-MSCs. The dissociation of the *KEAP1/Nrf2* complex, along with the expression of *phosphoinositide 3-kinase (PI3K)* and *protein kinase B (AKT)*, increased in the coculture group compared with the H_2_O_2_ treatment group (* p < 0.05). The expression levels of antioxidant genes increased in the cocultured group compared with those in the H_2_O_2_ treatment group (* *p* < 0.05), whereas the ROS levels decreased in the cocultured group (* *p* < 0.05). Additionally, both the expression of mitochondrial dynamics markers and the mitochondrial membrane potential increased when the cells were cocultured with PD-MSCs (* *p* < 0.05). PD-MSC cocultivation decreased the expression levels of lipoproteins (* *p* < 0.05). Finally, we confirmed that PD-MSCs promoted the expression of RPE-specific genes in H_2_O_2_-injured ARPE-19 cells (* p < 0.05). These findings suggest a new aspect of stem cell treatment for AMD induced by oxidative stress.

## 1. Introduction

The retinal pigment epithelium (RPE) plays a critical role in maintaining retinal homeostasis and supporting retinal cells such as photoreceptors. When the RPE is damaged, it can lead to various retinal diseases, including age-related macular degeneration (AMD). Given the high global incidence of AMD and its potential to cause blindness, proper functioning of the RPE is of paramount importance [1]. Injury to the RPE is caused by various factors, such as smoking and exposure to ultraviolet and visible light. These conditions generate reactive oxygen species (ROS) and induce oxidative stress in the retina [2]. AMD is classified into dry AMD and wet AMD. Dry AMD is a non-neovascular form of age-related macular degeneration that can progress to geographic atrophy (GA). Although wet AMD, which involves neovascularization, is treated with anti-VEGF therapy, there is currently no effective treatment for dry AMD [3,4]. These types of AMD commonly develop because of the accumulation of extracellular deposits called drusen, which subsequently leads to dysfunction of the retinal pigment epithelium (RPE) and loss of photoreceptors. Increased ROS levels disrupt the mitochondrial electron transport chain, causing electron leakage, lipid peroxidation, and cellular ferroptosis [5]. These events contribute to cholesterol accumulation and the formation of drusen between the RPE and choroid, further exacerbating retinal degeneration [6]. Drusen not only interferes with normal retinal function but also promotes chronic inflammation and oxidative stress and in some cases triggers pathological angiogenesis [7].

In retinal cells, particularly in the energy-demanding RPE and photoreceptors, mitophagy is critical for preserving mitochondrial function and preventing oxidative stress. Impaired mitophagy leads to the accumulation of damaged mitochondria, increased levels of reactive oxygen species (ROS), and mitochondrial dysfunction, all of which contribute to retinal degeneration [8]. Mitochondrial dysfunction plays a key role in AMD, and studies have shown that mitochondrial DNA damage in the RPE increases as AMD progresses, contributing to cellular dysfunction and retinal degeneration [9]. Consequently, mitochondrial injury plays a role in oxidative stress and inflammation, which are key factors in drusen formation [10]. As a key component in retinal homeostasis, the RPE plays a critical role in maintaining visual function by supporting photoreceptors and processing metabolic waste in the retina. However, RPE cells are highly susceptible to oxidative stress because of constant exposure to high levels of metabolic activity and external factors such as ultraviolet (UV) radiation [11]. Oxidative stress occurs when there is an imbalance between reactive oxygen species (ROS) production and the antioxidant defense system of the cell, leading to cellular damage, inflammation, and, in severe cases, degenerative retinal diseases such as age-related macular degeneration (AMD).

Nuclear factor erythroid 2-related factor 2 (Nrf2) is a key transcription factor that regulates cellular defense against oxidative stress. Under normal conditions, Nrf2 is kept in the cytoplasm by the repressor Kelch-like EDH-associated protein 1 (Keap1) [12]. However, in response to oxidative stress, Nrf2 dissociates from Keap1 and is translocated to the nucleus, where it binds to antioxidant response elements (AREs) in the promoter regions of various antioxidant genes [13]. This activation induces the expression of several protective enzymes, including superoxide dismutase (SOD), catalase (CAT), and heme oxygenase-1 (HO-1), which alleviate oxidative damage and restore cellular redox balance. In RPE cells, the activation of Nrf2 is particularly important for protection against oxidative damage, which is a major contributor to the pathogenesis of retinal degenerative diseases [14]. By enhancing the antioxidant response, Nrf2 plays a protective role in maintaining the functional integrity of RPE cells and consequently preserving visual function. Given the central role of oxidative stress in retinal diseases, understanding the regulation of Nrf2 in RPE cells may provide valuable insights into potential therapeutic strategies for preventing or treating conditions such as AMD [15].

Given the vulnerability of RPE cells to oxidative stress in AMD, therapeutic approaches targeting oxidative damage and inflammation are critical [16]. One such approach involves the use of pigment epithelium-derived factor (PEDF), which is identified in conditioned medium from fetal human RPE cell cultures [17]. PEDF can protect neurons from oxidative stress and glutamate toxicity, and in retinal cells, it can increase the expression of antioxidant proteins in AMD patients. Additionally, PEDF can inhibit choroidal neovascularization (CNV) associated with AMD because of its antiangiogenic effects [18]. PEDF plays a role in maintaining the structural stability of photoreceptors through the secretion by RPE, which inhibits the inflammatory response by regulating NF-κB pathways and induces the apoptosis of neovascular endothelial cells through the Fas/FasL pathway, confirming its therapeutic potential for AMD [19]. In our previous study, we reported that PD-MSCs promoted the recovery of retinal layers in H_2_O_2_-induced rat retinas [20]. In the present study, we aimed to compare the therapeutic efficacy of PD-MSCs with that of PEDF, a factor with known therapeutic potential, and to investigate whether PD-MSCs can activate Nrf2 in H_2_O_2_-damaged ARPE-19 cells, thereby exerting antioxidative effects.

## 2. Materials and Methods

### 2.1. Cell Culture

Placentas were collected from the chorionic plate of healthy women (37 gestational weeks) by the Institutional Review Board of CHA Gangnam Medical Center, Seoul, Republic of Korea (IRBs 07–18). PD-MSCs were isolated and incubated as previously described in modified minimal essential medium (MEM; HyClone, Logan, UT, USA) supplemented with 10% fetal bovine serum (FBS; Gibco, Carlsbad, CA, USA), 1% penicillin/streptomycin (P/S; Invitrogen, Carlsbad, CA, USA), 25 ng/mL human fibroblast growth factor-4 (FGF-4; PeproTech, Rocky Hill, NJ, USA), and 1 µg/mL heparin (Sigma-Aldrich, St. Louis, MO, USA) [21]. Human retinal pigment epithelium-derived ARPE-19 cells (ATCC, Manassas, VA, USA) were maintained in Dulbecco’s Modified Eagle Medium/Nutrient Mixture F-12 (DMEM/F-12; Gibco, Carlsbad, CA, USA) supplemented with 10% FBS (Gibco, Carlsbad, CA, USA) and 1% P/S (Invitrogen™, Carlsbad, CA, USA). The cells were cultured at 5% CO_2_ and 37 °C.

### 2.2. In Vitro Coculture System

To analyze the effects of PD-MSCs on oxidative stress induced in ARPE-19 cells (CRL-2302, ATCC, Manassas, VA, USA), ARPE-19 cells were treated with hydrogen peroxide (H_2_O_2_; 200 µM; Sigma-Aldrich, St. Louis, MO, USA) for 2 h and cocultured with naïve PD-MSCs (5 × 10^3^ cells/cm^2^) in 8-µm pore Transwell inserts (Corning, NY, USA) in α-MEM (HyClone, Logan, UT, USA) supplemented with 1% P/S (Invitrogen™, Carlsbad, CA, USA) for 24 h at 5% CO_2_ and 37 °C. To analyze the expression patterns of each signaling factor upon PEDF treatment, we treated ARPE-19 cells with recombinant PEDF (10 ng/mL; Peprotech, Carlsbad, CA, USA).

### 2.3. RNA Isolation and Quantitative Real-Time Polymerase Chain Reaction

Total RNA was extracted from ARPE-19 cells using TRIzol LS (Invitrogen™, Carlsbad, CA, USA) according to the manufacturer’s method. cDNA was synthesized using SuperScript III reverse transcriptase (Invitrogen™, Carlsbad, CA, USA). qRT–PCR was performed on a CFX Connect™ Real-Time System (Bio-Rad, Hercules, CA, USA) with primers (Supplementary Materials) and SYBR Green PCR master mix (Roche, Basel, Switzerland). Gene expression was quantified by the 2^−∆∆CT^ method, and all the data were analyzed in triplicate. Each sample was examined in triplicate, with human GAPDH as the internal control for standardization.

### 2.4. Protein Isolation and Western Blot

The samples were lysed in lysis buffer (Sigma-Aldrich, USA) supplemented with a phosphatase inhibitor (AG Scientific, San Diego, CA, USA) and a protease inhibitor cocktail (Roche, Basel, Switzerland). Protein lysates were separated by sodium dodecyl sulfate–polyacrylamide gel electrophoresis (SDS–PAGE) and transferred to PVDF membranes (Bio-Rad, Hercules, CA, USA). The following primary antibodies were used: anti-RDH11 (1:1000, bs-6214R, Bioss, Woburn, MA, USA), anti-RPE65 (1:1000, MA1-16578, Invitrogen™, Carlsbad, CA, USA), anti-HMOX1 (1:1000, NBP1-97507, Novus Biologicals, Centennial, CO, USA), anti-SOD1 (1:1000, 4266S, Cell Signaling Technology, Danvers, MA, USA), anti-catalase (1:1000, ab52477, Abcam, Cambridge, UK), anti-phospho DRP1 (1:1000, PA5-64821, Invitrogen™, Carlsbad, CA, USA), anti-OPA1 (ab157457, Abcam, Cambridge, UK), anti-ABCA1 (1:1000, NB400-105, NOVUS, Centennial, CO, USA), anti-ApoE (1:1000, A0304, ABclonal, Woburn, MA, USA), anti-PI3K p110α (1:1000, 4255s, Cell Signaling Technology, Danvers, MA, USA), anti-phospho AKT (1:1000, 9271S, Cell Signaling Technology, Danvers, MA, USA), anti-total AKT (1:1000, 9272S, Cell Signaling Technology, Danvers, MA, USA), anti-phospho Nrf2 (1:1000, BS2013R, Bioss, Woburn, MA, USA), anti-total Nrf2 (1:1000, BS1074R, Bioss, Woburn, MA, USA), anti-KEAP1 (1:1000, 8047S, Cell Signaling Technology, Danvers, MA, USA) and anti-GAPDH (1:1000, LF-PA0018, AbFrontier, Seoul, Republic of Korea). The following secondary antibodies were used: anti-HRP-conjugated mouse IgG (1:5000; 7076S; Cell Signaling Technology, Danvers, MA, USA) and anti-HRP-conjugated rabbit IgG (1:5000; 7074S; Cell Signaling Technology, Danvers, MA, USA). Each band was subjected to chemiluminescence detection using ECL reagent (Bio-Rad, Hercules, CA, USA) and quantified using ImageJ software (NIH, Bethesda, MD, USA). The images were analyzed in ImageJ 1.54g (Java 1.8.0_345, 64-bit).

### 2.5. Enzyme-Linked Immunosorbent Assay (ELISA)

Pigment epithelium-derived factor (*PEDF*) (Abcam, Waltham, MA, USA), superoxide dismutase 1 (*SOD1*) (Abcam, Waltham, MA, USA), and catalase (MyBioSource, San Diego, CA, USA) activities in the cell culture supernatant from ARPE-19 cells cocultured with PD-MSCs using a Transwell system were analyzed using ELISA kits in accordance with the manufacturer’s instructions. The same amount of sample was placed on specific antibody-coated plates. The appropriate horseradish peroxidase (HRP)-conjugates were then added to each well, and the wells were then incubated at 37 °C. A microplate reader was used to measure the antibody activity after the substrates had been introduced and allowed to develop in the dark (BioTek, Winooski, VT, USA).

### 2.6. Immunofluorescence Staining

ARPE-19 cells were fixed with 4% paraformaldehyde (eLbio, Seoul, Republic of Korea). Next, 1× phosphate-buffered saline (PBS) was used to wash the fixed cells. The blocking solution (DAKO, Glostrup, Denmark) was applied to the cells for an hour at room temperature, and the antibody (1:300) was applied to each tissue sample overnight in a 4 °C cold room. After three rounds of washing with 1× PBS at room temperature for 5 min each, the cells were exposed to a secondary antibody (1:250) for one hour at room temperature. The samples were then washed three times for five minutes in 1× PBS at room temperature. Afterward, the cells were mounted in mounting media supplemented with DAPI (VECTASHIELD^®^, Burlingame, CA, USA). The following primary antibodies were used: anti-ABCA1 (1:250, NB400-105, NOVUS Centennial, CO, USA), Anti-cleaved Caspase3 (1:250, 9661, Cell Signaling Technology, Danvers, MA, USA), anti-RPE65 (1:250, MA1-16578, Invitrogen™, USA), anti-OPA1 (1:250, ab157457, Abcam, Cambridge, UK), anti-phospho Nrf2 (1:250, BS2013R, Bioss Woburn, MA, USA) and anti-KEAP1 (1:250, 8047S, Cell Signaling Technology, Danvers, MA, USA). The following secondary antibodies were used: Alexa Fluor 488, Alexa Fluor 594 (1:100, Invitrogen™, Grand Island, USA). A fluorescence microscope was used to look at the created slides. The entirety of each slide was examined, and a representative image was taken. ImageJ was used to analyze the cells (ImageJ 1.54g (Java 1.8.0_345, 64-bit)).

### 2.7. MitoSOX/MitoTracker

After the cells were harvested, they were washed in 1× PBS (eLbio, Seoul, Republic of Korea). Afterward, the cells were incubated with 1.5 μM MitoSOX^TM^ (superoxide staining, red signals; Invitrogen™, Grand Island, USA) and 50 nM MitoTracker^TM^ (mitochondrial staining, green signals; Invitrogen™, Grand Island, USA) for 40 min at 37 °C. Afterward, the cells were mounted in mounting media supplemented with DAPI (VECTASHIELD^®^, Burlingame, CA, USA). The cells were observed via confocal microscopy (Zeiss 780; Zeiss, Oberkochen, Germany) at 40× magnification, and images of randomized areas of all the slides were captured. We quantified 29~35 cells (6~7 cells with 5 images).

### 2.8. JC-1 Staining

ARPE-19 cells from the control, H_2_O_2_, and PD-MSC coculture groups were treated with 20 µM JC-1 solution (T-3168; Invitrogen™, Grand Island, USA) and incubated for 10 min in the dark. The fluorescence top reading was subsequently performed by a microplate reader (Infinite^®^ 200 PRO, Tecan, Männedorf, Switzerland). The fluorescence intensity of the monomers was measured at 514 nm (excitation) and 529 nm (emission), and that of the aggregates was measured at 585 nm (excitation) and 590 nm (emission). The cells treated with a 5 µM JC-1 solution were subjected to confocal scanning microscopy (Zeiss LSM880; Carl Zeiss, Oberkochen, Germany).

### 2.9. TBARS Assay

To measure the extent of lipid peroxidation, the formation of malondialdehyde (MDA) in the ARPE-19 cell supernatant was assessed using a TBARS assay (KGE013, R&D Systems, Minneapolis, MN, USA).

### 2.10. BODIPY Staining

ARPE-19 cells were fixed with 4% paraformaldehyde for 10 min. The samples were rinsed three times for 5 min each with precooled 1× PBS. The samples were incubated with a 10 μg/mL BODIPY^®^ 505/515 (Thermo Fisher Scientific, Waltham, MA, USA) solution for 30 min at 37 °C. The samples were rinsed three times for 5 min each with precooled 1× PBS. Afterward, the samples were counterstained and mounted using mounting medium containing DAPI (VECTASHIELD^®^, Burlingame, CA, USA). Fluorescence microscopy (Zeiss Axiocam 506 color, Oberkochen, Germany) was used to observe the tissues at 40× magnification. All parts of each slide were observed, and representative images were captured.

### 2.11. Statistical Analysis

Each experiment was carried out two or three times. Basically, the experiments were performed three times; however, when the values showed large deviations from the mean, the results were presented based on two repetitions for statistical analysis. The data is expressed as the mean ± standard error of the mean (SEM). Tukey’s post hoc test was used after a one-way ANOVA for between-group comparisons of data obtained at various time points. PRISM 5.01 (GraphPad Software version 5.01; San Diego, CA, USA) was used to evaluate the data, and a p value less than 0.05 was considered to indicate statistical significance.

## 3. Results

### 3.1. PD-MSC Cocultivation Decreased Lipid Accumulation in ARPE-19 Cells Exposed to H_2_O_2_

**Figure 1:**
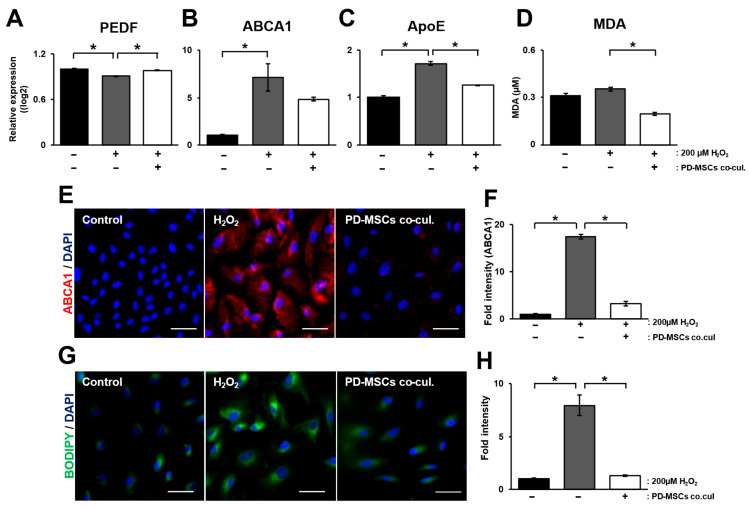
PD-MSCs cocultivation decreased lipid accumulation in ARPE-19 cells exposed to H_2_O_2_. (**A**) mRNA expression of PEDF in H_2_O_2_-treated ARPE-19 cells cocultured with PD-MSCs. mRNA expression of (**B**) ABCA1 and (**C**) ApoE in H_2_O_2_-treated ARPE-19 cells cocultured with PD-MSCs. (**D**) The concentration of malondialdehyde in cell culture supernatant in H_2_O_2_-treated ARPE-19 cells cocultured with PD-MSCs. (**E**) Representative images and (**F**) quantitative analysis of ABCA1 in H_2_O_2_-treated ARPE-19 cells cocultured with PD-MSCs. DAPI was used for counterstaining. Scale bar = 50 µm. (**G**) Representative images and (**H**) quantitative analysis of lipid droplet formation in H_2_O_2_-treated ARPE-19 cells cocultured with PD-MSCs using the BODIPY staining. DAPI was used for counterstaining. Scale bar = 50 µm. The data represent 2–3 independent experiments for each group and are expressed as the mean ± SEM. Statistical significance was determined by one-way ANOVA (* *p* < 0.05).

First, we analyzed the mRNA expression of PEDF in ARPE-19 cells cocultured with PD-MSCs. The coculture was conducted using a transwell system. To analyze mRNA levels specifically from ARPE-19 cells, we cultured and harvested the cells in the lower chamber of the transwell insert. H_2_O_2_ treatment significantly decreased PEDF mRNA levels compared with control, whereas PD-MSC coculture prevented this H_2_O_2_-induced decrease (Figure 1A; *p* < 0.05). Lipid metabolism in RPE cells plays a critical role in maintaining communication with photoreceptors. However, oxidative stress can disrupt lipid metabolism, thereby impairing the normal function of related regulatory factors [22]. The mRNA expression of ABCA1 and ApoE, which are related to lipoproteins, increased in the H_2_O_2_-treated group but significantly decreased in the PD-MSC coculture group (Figure 1B,C; *p* < 0.05). The level of malondialdehyde (MDA), a product of lipid peroxidation, increased in the H_2_O_2_-treated group but significantly decreased in the PD-MSC coculture group (Figure 1D; *p* < 0.05). Representative images revealed the same trend as that of the mRNA expression of ABCA1 (Figure 1E,F; *p* < 0.05). To verify the formation of lipid droplets, we analyzed ARPE-19 cells by BODIPY staining. The increase in lipid formation induced by H_2_O_2_ treatment in the ARPE-19 cells dramatically decreased in the PD-MSC coculture group (Figure 1G,H; *p* < 0.05). These results indicate that PD-MSCs inhibit the formation of lipoproteins in H_2_O_2—_injured ARPE-19 cells.

### 3.2. PD-MSC Cocultivation Ameliorates Oxidative Stress by Enhancing the Accumulation of Antioxidants in ARPE-19 Cells Exposed to H_2_O_2_

**Figure 2:**
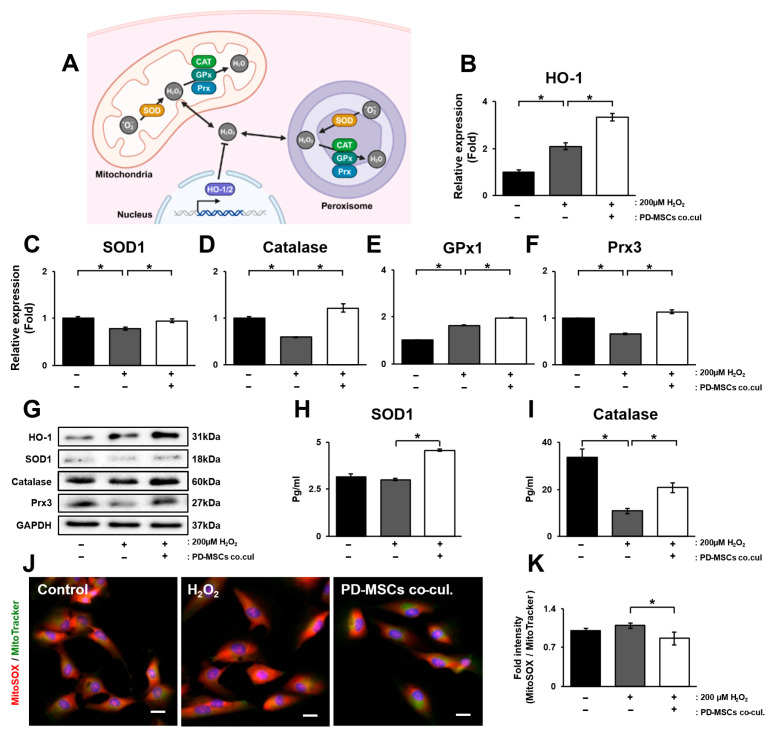
PD-MSCs cocultivation ameliorates oxidative stress by enhancing antioxidant accumulation in ARPE-19 cells exposed to H_2_O_2_. (**A**) Schematic of antioxidative mechanism of antioxidant enzymes (HO-1/2, SOD, catalase, GPx and Prx). (**B**–**F**) mRNA expression of antioxidants in H_2_O_2_-treated ARPE-19 cells cocultured with PD-MSCs. (**G**) Western blot analysis of antioxidant enzymes in cell lysates of H_2_O_2_-treated ARPE-19 cells cocultured with PD-MSCs. The concentration of (**H**) SOD1 and (**I**) catalase in cell culture supernatant of H_2_O_2_-treated ARPE-19 cells cocultured with PD-MSCs. (**J**) Representative images and (**K**) quantitative analysis of mitochondrial ROS levels in H_2_O_2_-treated ARPE-19 cells cocultured with PD-MSCs using the MitoSOX assay. DAPI was used for counterstaining. Scale bar = 100 μm. The data represent 2–3 independent experiments for each group and are expressed as the mean ± SEM. Statistical significance was determined by one-way ANOVA (* *p* < 0.05).

Antioxidative effects are critical for maintaining retinal homeostasis in the context of oxidative stress. In particular, superoxide dismutase (SOD), catalase (CAT), glutathione peroxidase (GPx) and peroxiredoxin (Prx) are key antioxidant enzymes in mitochondria and peroxisomes (Figure 2A). To analyze mRNA levels specifically from ARPE-19 cells, we cultured and harvested the cells in the lower chamber of the transwell insert. Compared with those in the H_2_O_2_ treatment group, the mRNA expression of HO-1, SOD1, catalase, GPx1 and Prx3 significantly increased in the PD-MSC coculture group (Figure 2B–F; *p* < 0.05).

The protein levels of HO-1, SOD1, catalase and Prx3 showed the same trend as the mRNA expression (Figure 2G). To confirm endogenous SOD1 and catalase levels in ARPE-19 cells, we analyzed the culture supernatants of ARPE-19 cells. These parameters decreased in the H_2_O_2_-treated group but significantly increased in the PD-MSC coculture group (Figure 2H,I; *p* < 0.05). To analyze whether PD-MSCs affect mitochondrial oxidative stress, mitochondrial superoxide ROS levels were detected in ARPE-19 cells using MitoSOX staining. The activity of mitochondrial ROS increased in the H_2_O_2_-treated group but decreased in the PD-MSC coculture group (Figure 2J,K; *p* < 0.05). These results suggest that PD-MSCs ameliorate oxidative stress by enhancing antioxidant effects.

### 3.3. PD-MSC Cocultivation Enhances Mitochondrial Membrane Potential by Modulating Mitochondrial Dynamics in ARPE-19 Cells Exposed to H_2_O_2_

**Figure 3:**
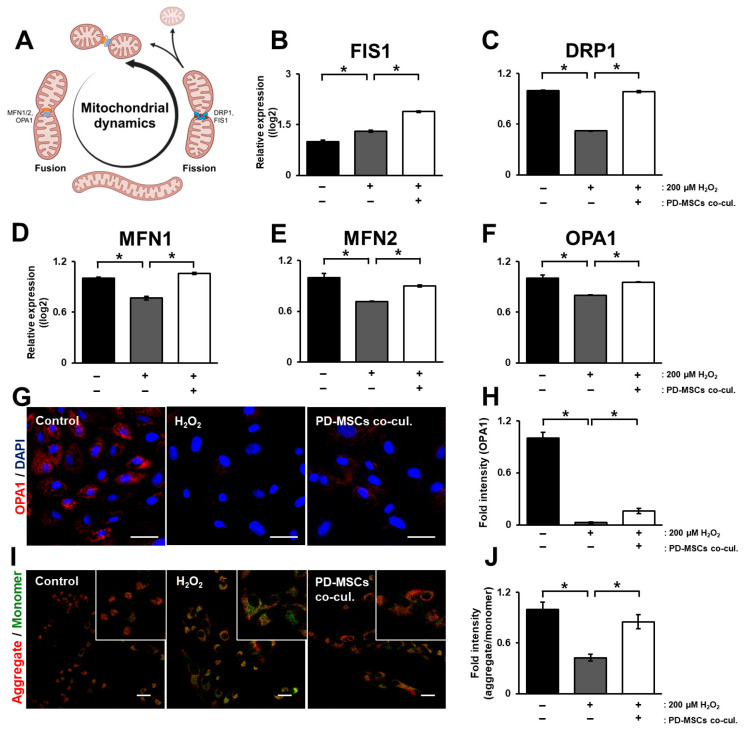
PD-MSCs cocultivation enhances mitochondrial membrane potential by modulating mitochondrial dynamics in ARPE-19 exposed to H_2_O_2_. (**A**) Schematic of mitochondrial dynamics between mitochondrial fission and fusion. (**B**,**C**) mRNA expression of mitochondrial fission markers (FIS1, DRP1) levels in H_2_O_2_-treated ARPE-19 cells cocultured with PD-MSCs. (**D**–**F**) mRNA expression of mitochondrial fusion markers (MFN1, MFN2, and OPA1 levels in H_2_O_2_-treated ARPE-19 cells cocultured with PD-MSCs. (**G**) Representative images and (**H**) quantitative analysis of OPA1 (red) in H_2_O_2-_-treated ARPE-19 cells cocultured with PD-MSCs. DAPI (blue) was used for counterstaining. Scale bar = 50 µm. (**I**) Representative images and (**J**) quantitative analysis of mitochondrial membrane in H_2_O_2_-treated ARPE-19 cells cocultured with PD-MSCs using JC-1 fluorescence assay. Scale bar = 25 μm. The data represent 2–3 independent experiments for each group and are expressed as the mean ± SEM. Statistical significance was determined by one-way ANOVA (* *p* < 0.05).

Mitochondrial dynamics, which are important mechanisms of the mitochondrial cycle, include “fusion” and “fission”. Mitofusin 1/2 (MFN1/2) and OPA1 (optic atrophy 1) are related to mitochondrial fusion [23]. Dynamin-related protein 1 (DRP1) and fission-protein 1 (FIS1) are related to mitochondrial fission (Figure 3A). The mRNA expression of FIS1 increased in the H_2_O_2_-treated group but increased more in the PD-MSC coculture group (Figure 3B; *p* < 0.05). The mRNA expression of DRP1, MFN1/2 and OPA1 decreased in the H_2_O_2_-treated group but significantly increased in the PD-MSC coculture group (Figure 3C–F; *p* < 0.05). Representative images revealed the same trend as that of the mRNA expression of OPA1 (Figure 3G,H; *p* < 0.05). The mitochondrial membrane potential is a key indicator of mitochondrial energy storage and activity [24]. To analyze whether PD-MSCs increase the mitochondrial membrane potential, we conducted JC-1 staining in ARPE-19 cells. The ratio of aggregated and monomeric JC-1 decreased in the H_2_O_2_-treated group but significantly increased in the PD-MSC coculture group (Figure 3I,J; *p* < 0.05).

### 3.4. PD-MSC Cocultivation Improves the Visual Cycle in ARPE-19 Cells Exposed to H_2_O_2_

**Figure 4:**
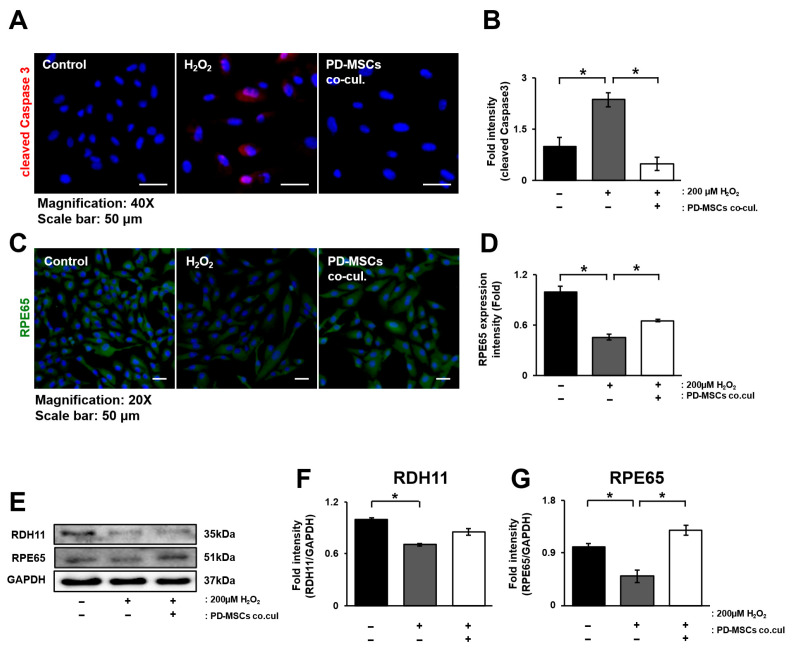
PD-MSC cocultivation improves visual cycles in ARPE-19 cells exposed to H_2_O_2_. (**A**) Representative images and quantitative analysis of (**B**) cleaved caspase3 (red) in H_2_O_2_-treated ARPE-19 cells cocultured with PD-MSCs. (**C**) Representative images and quantitative analysis of (**D**) RPE65 (green) in H_2_O_2_-treated ARPE-19 cells cocultured with PD-MSCs. DAPI (blue) was used for counterstaining. Scale bar = 50 µm. (**E**) Western blot analysis and (**F**,**G**) quantification of RPE-specific markers (RDH11 and RPE65, respectively) in cell lysates of H_2_O_2_-treated ARPE-19 cells cocultured with PD-MSCs. The data represent 2–3 independent experiments for each group and are expressed as the mean ± SEM. Statistical significance was determined by one-way ANOVA (* *p* < 0.05).

The visual cycle between the retinal pigment epithelium (RPE) and photoreceptors is critical for the visual process related to vitamin A [25]. RPE cells exposed to oxidative stress undergo apoptosis and exhibit downregulation of the visual cycle. To examine whether coculture with PD-MSCs could modulate these changes, immunofluorescence staining for caspase-3 and RPE65 was performed [26]. The expression of cleaved caspase 3 significantly increased in the H_2_O_2_-treated group and decreased in the PD-MSC coculture group. However, the expression of RPE65 showed the opposite trend (Figure 4A–D; *p* < 0.05). The protein levels of RDH11 and RPE65 decreased in the H_2_O_2_-treated group but increased in the PD-MSC coculture group (Figure 4E–G; *p* < 0.05).

### 3.5. PD-MSC Cocultivation Activates the Nrf2 Signaling Pathway in ARPE-19 Cells Exposed to H_2_O_2_

**Figure 5:**
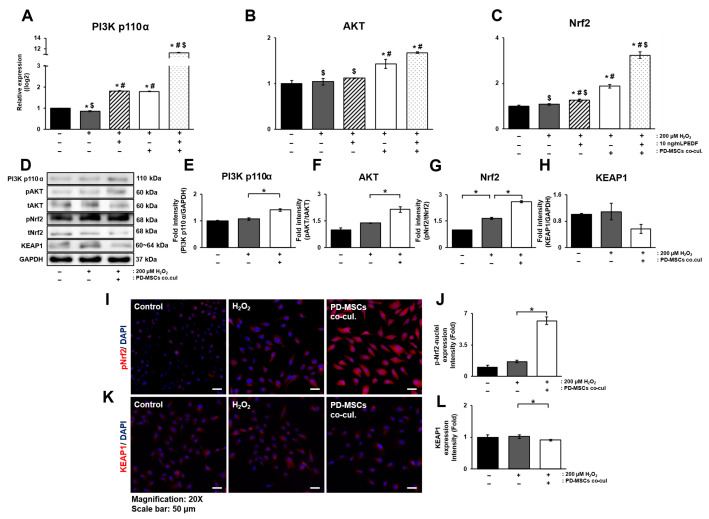
PD-MSC cocultivation activates the Nrf2 signaling pathway in ARPE-19 cells exposed to H_2_O_2_. mRNA expression of (**A**) PI3K p110a, (**B**) AKT and (**C**) Nrf2 levels in H_2_O_2_-treated ARPE-19 cells treated with 10 ng/mL PEDF, cocultured PD-MSCs or their combination. (**D**) Western blot analysis of antioxidant enzymes in cell lysates of H_2_O_2_-treated ARPE-19 cells cocultured with PD-MSCs. Quantification of (**E**) PI3K p110a, (**F**) AKT, (**G**) Nrf2 and (**H**) KEAP1 in cell lysates of H_2_O_2_-treated ARPE-19 cells cocultured with PD-MSCs. Quantitative analysis and representative images of (**I**,**J**) phospho-Nrf2 and (**K**,**L**) KEAP1 in H_2_O_2_-treated ARPE-19 cells cocultured with 10 ng/mL PEDF, PD-MSCs or their combination. The negative correlation between phospho-Nrf2 and KEAP1 was determined by the intensity of IF staining. DAPI was used for counterstaining. Scale bar = 50 µm. The data represent 2–3 independent experiments for each group and are expressed as the mean ± SEM. Statistical significance was determined by one-way ANOVA (* *p* < 0.05 vs. control, # *p* < 0.05 vs. H_2_O_2_ treatment, $ *p* < 0.05 vs. PD-MSCs cocultivation).

To analyze the activation of Nrf2, a key regulator of antioxidant enzymes, the PI3K/AKT signaling pathway, which is an upstream regulatory pathway of Nrf2, was evaluated at the level of mRNA expression. Additionally, we investigated whether PEDF could activate this mechanism. The mRNA expression of PI3K p110α decreased in the H_2_O_2_-treated group but significantly increased in the PD-MSC coculture group. PEDF treatment alone significantly increased the expression of PI3K p110α compared with that in the H_2_O_2_-treated group, but the difference was not substantial compared with that in the PD-MSC group. However, the combination treatment of PD-MSCs and PEDF noticeably increased the expression of PI3K p110α (Figure 5A; *p* < 0.05). The expression of AKT did not increase significantly with PEDF treatment alone. However, it was significantly upregulated in cells cocultured with PD-MSCs alone and in combination with PD-MSCs and PEDF (Figure 5B; *p* < 0.05). The mRNA expression of Nrf2 showed the same trend as that of AKT (Figure 5C; *p* < 0.05). The protein levels of PI3K p110α, AKT and Nrf2 decreased in the H_2_O_2_-treated group but increased in the PD-MSC coculture group. In addition, KEAP1 expression was elevated in the H_2_O_2_-treated group but tended to decrease when the cells were cocultured with PD-MSCs, and the opposite pattern was observed for Nrf2 expression (Figure 5D–H; *p* < 0.05). To determine the correlation between Nrf2 activation and that of its suppressor KEAP1, immunostaining was performed. The expression of Nrf2 showed a slight, though not statistically significant, increase following H_2_O_2_ treatment. Subsequently, coculture with PD-MSCs led to a significant elevation in Nrf2 expression. In the case of KEAP1, its expression did not show a notable change after H_2_O_2_ treatment, but exhibited a slight decrease when cocultured with PD-MSCs. KEAP1 expression was inversely related to Nrf2 expression in the PD-MSC coculture condition (Figure 5I–L).

## 4. Discussion

In this study, we aimed to determine whether placenta-derived mesenchymal stem cells (PD-MSCs) could restore H_2_O_2_-injured retinal pigment epithelium (RPE) cells by activating Nrf2-mediated antioxidant pathways. Our findings demonstrate that PD-MSCs contribute to the recovery of several cellular functions, including the suppression of lipoprotein formation (Figure 1), upregulation of antioxidant activity (Figure 2), restoration of mitochondrial function (Figure 3), regulation of visual cycle genes (Figure 4), and activation of the Nrf2 signaling pathway (Figure 5).

This study focused on three aspects: the effects of PD-MSCs, oxidative stress and mitochondrial function, and the upregulation of visual cycle-related genes. In our previous work, PEDF-overexpressing PD-MSCs promoted recovery of the retinal layer in an H_2_O_2_-injured rat model [20,27]. The PI3K/AKT pathway is one of the signaling cascades that regulate the expression of Nrf2, and its analysis can provide indirect evidence for the modulation of Nrf2 expression. In this study, we analyzed the effects of naïve PD-MSCs on Nrf2 pathway activation in H_2_O_2_-injured ARPE-19 cells. Coculture with PD-MSCs led to greater expression of PI3K p110α, AKT, and Nrf2, suggesting that PD-MSCs play a direct role in activating the Nrf2 signaling pathway. This may be due to the broad cytokine secretory capacity of PD-MSCs. Furthermore, coculture with PD-MSCs under oxidative stress conditions led to even greater increases in PI3K, AKT, and Nrf2 mRNA expression, indicating a potential synergistic effect that warrants further investigation. Reactive oxygen species (ROS) are a major cause of RPE damage in age-related macular degeneration (AMD), and H_2_O_2_ induces oxidative stress, organelle dysfunction, cell death, and visual impairment [28]. Nrf2 is a key transcription factor that regulates antioxidant responses, and its activation is essential for protecting cells from oxidative stress. In our study, PD-MSC coculture increased the expression of antioxidant markers, which is consistent with the activation of the Nrf2 pathway. Although HO-1 and GPx1 were also upregulated in the H_2_O_2_-treated group, this may reflect an endogenous self-defense response [29,30]. We also analyzed the protein levels of SOD1 and catalase in the culture medium of ARPE-19 cells using ELISA (Figure 2H,I). However, in the case of coculture with PD-MSCs, it is possible that factors secreted from PD-MSCs were also detected, since the culture media of both cell types could be exchanged through the transwell system. Nevertheless, the results showed a similar trend to the changes observed at the mRNA level. In future experiments, it will be necessary to perform this assay under conditions that exclude such cross-detection. The marginal increase in MitoSOX levels indicates that H_2_O_2_-induced injury had only a mild effect on mitochondrial superoxide generation. Nevertheless, as further described below, we also evaluated mitochondrial membrane potential using JC-1 staining to determine whether such injury could influence mitochondrial function. The 2 h treatment with 200 µM H_2_O_2_ used in this study likely induced relatively mild oxidative damage, which may not have fully suppressed intrinsic antioxidant mechanisms (Figure 2).

Excess ROS also impair mitochondrial electron transport chains and cause electron leakage and lipid peroxidation, leading to cholesterol accumulation and ultimately drusen formation—hallmarks of dry AMD. Drusen formation, in turn, promotes inflammatory responses and pathological neovascularization [31]. PD-MSC treatment increased the expression of mitochondrial fusion markers (MFN1, MFN2, and OPA1) and restored the mitochondrial membrane potential (Figure 3). However, the expression patterns of the mitochondrial fission markers FIS1 and DRP1 were inconsistent, indicating the need for further studies to evaluate the role of mitophagy, which is closely linked to mitochondrial fission [32]. In addition, coculture with PD-MSCs suppressed the expression of lipoprotein-related genes associated with drusen formation and significantly reduced the expression of malondialdehyde (MDA), a lipid peroxidation byproduct, suggesting reduced oxidative stress (Figure 1). JC-1 staining confirmed the recovery of the mitochondrial membrane potential in PD-MSC-treated cells (Figure 3), further supporting the improvement in mitochondrial function.

To assess the recovery of visual function in vitro, as an alternative to confirming improvements in visual acuity in animal models or in humans, we analyzed factors related to the visual cycle. The visual cycle refers to the retinoid metabolism that occurs between photoreceptors and the retinal pigment epithelium (RPE) [25,33], which is often dysregulated in many ocular diseases. Moreover, gene mutations in vision cycle proteins are associated with various retinal disorders [34]. In particular, the visual cycle in the RPE is mediated by LRAT (lecithin retinol acyltransferase), RPE65, and RDH5/11 (retinol dehydrogenases) [35]. To investigate the effects of PD-MSC coculture on cell death and the visual cycle and to determine their correlation, we performed double staining for cleaved caspase-3 and RPE65 (Figure 4A–D). This analysis revealed that RPE65 expression increases in contrast to apoptosis, indicating that PD-MSCs reduce apoptosis and increase the expression of visual cycle-related factors.

Currently available treatments for dry AMD primarily aim to slow the progression of geographic atrophy rather than regenerating damaged tissue [36]. Recently, complement inhibitors have been approved by the FDA for this purpose, but they show limited efficacy in improving visual function and raise concerns about long-term outcomes and cost [37]. In this context, stem cell-based therapies have emerged as promising alternatives because of their regenerative potential in damaged retinal tissues. Conventional approaches typically focus on stem cell differentiation and the replacement of damaged cells. However, these strategies face major limitations, including low engraftment, immune rejection, and tumorigenic risks [38]. In contrast, mesenchymal stem cells (MSCs) are known for their immunomodulatory, antioxidant, anti-inflammatory, and tissue-regenerative properties [39]. MSCs secrete neurotrophic and antiangiogenic factors, which may contribute to the repair and regeneration of retinal layers.

Human placenta-derived mesenchymal stem cells (PD-MSCs), which are derived from fetal tissue, offer several advantages over other mesenchymal stem cells (MSCs) [21]. These advantages include strong immunosuppressive properties and an enhanced proliferative capacity compared with those of other types of MSCs. As a result, fewer passages are needed for PD-MSCs to reach a substantial cell count, reducing the risk of cellular senescence [37]. Studies have also reported that exosomes or the culture medium of MSCs enhance antioxidant effects and reduce retinal toxicity in AMD models [40,41]. Compared with other MSCs, PD-MSCs exhibit superior immunomodulatory capacity and enhanced self-renewal ability. For example, PD-MSCs have been reported to secrete factors such as brain-derived neurotrophic factor (BDNF), nerve growth factor (NGF), and neurotrophin-3 (NT-3) in hypoxia-damaged rat retinal neurons, promoting neuronal regeneration and axon regeneration through paracrine effects [42]. Additionally, in a study involving optic nerve compression, significant increases in the expression of GAP43, Tuj1, and GFAP were observed in groups treated with injections of PD-MSCs [43].

PEDF is a well-known antiangiogenic, anti-inflammatory, and neuroprotective factor and has been studied as a therapeutic agent for neovascular retinal diseases [44]. A comparative analysis of PEDF and PD-MSCs may provide insights into their respective advantages in AMD treatment. MSCs promote retinal layer restoration and regeneration through the secretion of neurotrophic and antiangiogenic factors, potentially complementing the effects of PEDF on neuroprotection and angiogenesis inhibition. Despite the potential of MSCs for ocular disease therapy, issues regarding cell survival rate and self-renewal capacity remain a challenge. Thus, research on the treatment of retinal degenerative diseases using MSCs should continue as the sustained survival and enhancement of the secretory capacity of MSCs remain important challenges.

Based on our findings, we demonstrate that PD-MSC-derived cytokines exert beneficial paracrine effects on H_2_O_2_-injured RPE cells, contributing to cell recovery. And we can also discuss the administration routes of MSCs. Since MSCs exert paracrine effects, intravitreal injections can be used. This route is more stable than subretinal administration, which is commonly applied for differentiated RPE cells [33].

Collectively, our results indicate that PD-MSCs suppress ROS production and mitigate oxidative stress in RPE cells. However, the oxidative damage model used in this study was relatively mild, allowing for the persistence of intrinsic defense mechanisms [45]. In addition, as this study was performed exclusively in vitro, it is limited in its ability to fully recapitulate the effects observed in vivo. Accordingly, future studies should carefully examine the optimal conditions for PD-MSC administration and disease induction in in vivo models. Because retinal diseases such as AMD progress in stages, examining the therapeutic effects of PD-MSCs across a broader spectrum of oxidative injury severity is important.

In conclusion, we demonstrated that PD-MSCs promote the recovery of H_2_O_2_-injured ARPE-19 cells through Nrf2-mediated antioxidant mechanisms. Nevertheless, the direct role of PEDF in this process remains unclear and requires further investigation under various experimental conditions. Additional studies are needed to elucidate the molecular mechanisms by which PEDF modulates retinal repair and to explore its potential synergistic effects with PD-MSCs.

## 5. Conclusions

**Figure 6:**
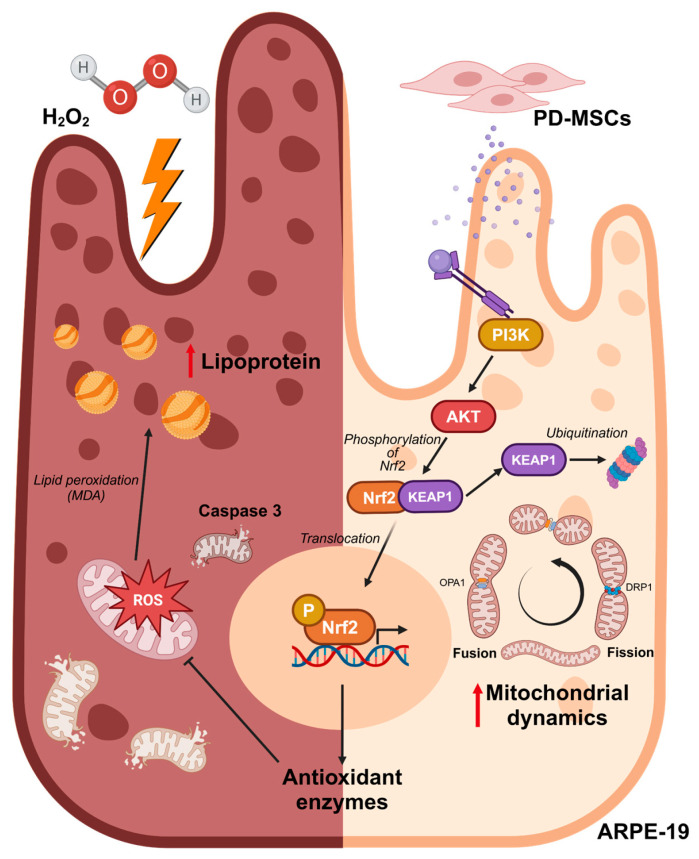
Summary illustration of the therapeutic effects of PD-MSCs on H_2_O_2_-injured RPE by increasing antioxidant enzymes via the Nrf2 pathway. PD-MSCs activate Nrf2 through the PI3K/AKT signaling pathway, promoting the expression of antioxidant enzymes. Mitochondrial dynamics are regulated, leading to reduced mitochondrial ROS levels and stabilized mitochondrial membrane potential. RPE regeneration is enhanced with decreased lipoprotein accumulation and reduced apoptosis.

H_2_O_2_ increases mitochondrial ROS levels in ARPE-19 cells, leading to mitochondrial dysfunction. Additionally, it induces apoptosis through lipid peroxidation and accumulation. PD-MSCs activate Nrf2, increasing the expression of antioxidants, improving mitochondrial function, and reducing lipid peroxidation and accumulation. PD-MSCs can regulate Nrf2 through the PI3K/AKT signaling pathway (Figure 6).

## Data Availability

The original contributions presented in this study are included in the article. Further inquiries can be directed to the corresponding author.

## Supplementary Materials

The following supporting information can be downloaded at: https://www.mdpi.com/article/10.3390/antiox14111279/s1. Supplementary materials; Figure S1: Melting curve of HO-1 by qRT-PCR; Figure S2: mRNA level of PEDF, ABCA1 and ApoE presented in a dot graph; Figure S3: mRNA level of HO-1, SOD1, CAT, GPx1 and Prx3 presented in a dot graph; Figure S4: Protein level of HO-1, SOD1, Catalase snd Prx3; Figure S5: mRNA level of FIS1, DRP1, MFN1, MFN2 and OPA1 presented in a dot graph; Figure S6: Protein level of RPE65 and RDH11; Figure S7: mRNA level of PI3K p110a, AKT and Nrf2 presented in a dot graph; Figure S8: Protein level of PI3K p110a, pAKT, tAKT, pNrf2, tNrf2 and KEAP1.

## Abbreviations

The following abbreviations are used in this manuscript:

AMD	Age-related macular degeneration
PD-MSCs	Placenta-derived mesenchymal stem cells
RPE	Retinal pigment epithelium
