# Supplementary material for: Nrf2 Activated by PD-MSCs Attenuates Oxidative Stress in a Hydrogen Peroxide-Injured Retinal Pigment Epithelial Cell Line
Source: Antioxidants (Basel). 2025 Oct 25;14(11):1279. doi: 10.3390/antiox14111279 (PMC12649114; doi:10.3390/antiox14111279)
Supplement: Supplementary file 1 [file antioxidants-14-01279-s001.zip › antioxidants-3885131-Supplementary Materials.pdf]

Supplementary materials

Gene	Primer	Annealing Temperature (℃)
HO-1	5'-ATGACACCAAGGACCAGAGC-3'	58.7
	5'-GTAAGGACCCATCGGAGAAGC-3'	59.2
HO-2	5'-ATGTCAGCGGAAGTGGAA-3'	55.2
	5'-GGGAGTTTCAGTGCTCGC-3'	57.5
SOD1	5'-AGTGCAGGGCATCATCAATTTCGAGCAG-3'	69.1
	5'-GATGCAATGGTCTCCTGAGAGTGAGATC-3'	63.2
Catalase	5'-TGCGGAGATTCAACACTGCC-3'	60
	5'- AGCAGGTGACTTCCATAGCA-3'	57.7
GPx1	5'-ACACCCAGATGAACGAGCTG-3'	59.1
	5'- CAAACTGGTTGCACGGGAAG-3'	59
Prx3	5'-CCCCAAGGAATGGCACTCAC-3'	60
	5'-ACGATGCCCTTTCAGCCTTC-3'	59.7
FIS1	5'-ATGGAGGCCGTGCTGAAC-3'	59.1
	5'-TCAGGATTTGGACTTGGA-3'	51.5
DRP1	5'-CACAGGAGGAGGTGGACAGC-3'	61.2
	5'-CGCCTCCTTCAGTGCGTGGT-3'	64.2
MFN1	5'-AGTAACAGGATTGGCGTCCG-3'	59.1
	5'-CGTTTCCTCCTATCATGGTCACC-3'	59.8
MFN2	5'-ATGCATCCCCACTTAAGCAC-3'	57.3
	5'-CCAGAGGGCAGAACTTTGTC-3'	57.5
OPA1	5'-GGCCAGCAAGATTAGCTACG-3'	57.8
	5'-ACAATGTCAGGCACAATCCA-3'	56.7
ABCA1	5'-AAGCACTTCCTCCGAGTCAA-3'	58
	5'- CTGTCCTTGGCCAGCTTTAG-3'	57.6
ApoE	5'-CTGCTCAGCTCCCAGGTC-3'	58.5
	5'-TTGTTCCTCCAGTTCCGATT-3'	55.8
PEDFR	5'-ACCATCCGGTGTGAGGGC-3'	60.7
	5'-TTCCCTGGTAACCATCACTCG-3'	58.5
PI3K p110α	5'-GGAGCCTGGAAGAGCCC-3'	58
	5'-CGTGGAGGCATTGTTCTGAT-3'	57.3
AKT	5'-GTCGCCTGCCCTTCTACAAC-3'	60.1
	5'-CACACGATACCGGCAAAGAA-3'	57.6
Nrf2	5'-GGTTGCCCACATTCCCAAATC-3'	59.1
	5'-CAAGTGACTGAAACGTAGCCG-3'	58.6
GAPDH	5'-GCACCGTCAAGGCTGAGAAC-3'	60.6
	5'-GTGGTGAAGACGCCAGTGGA-3'	61.4

Figure S1.

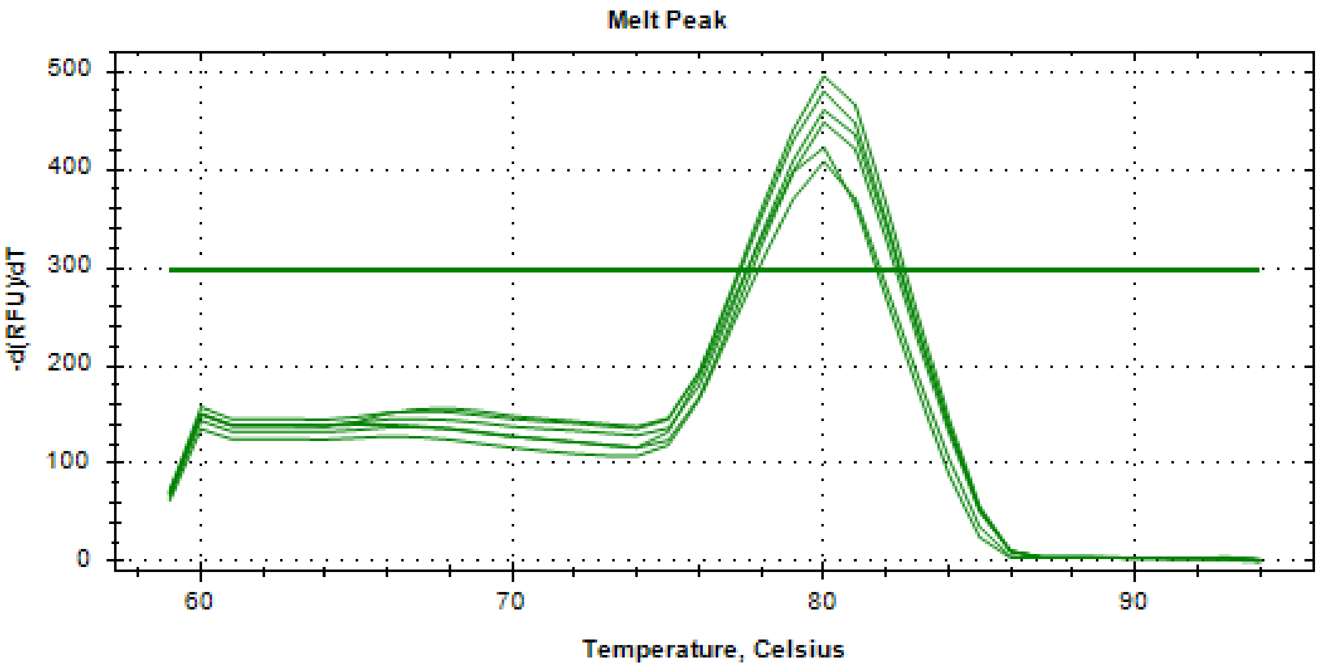

Melt Peak
500
400
300
200
100
0
-d(RFU)/dT
60
70
80
90
Temperature, Celsius

Figure S2.

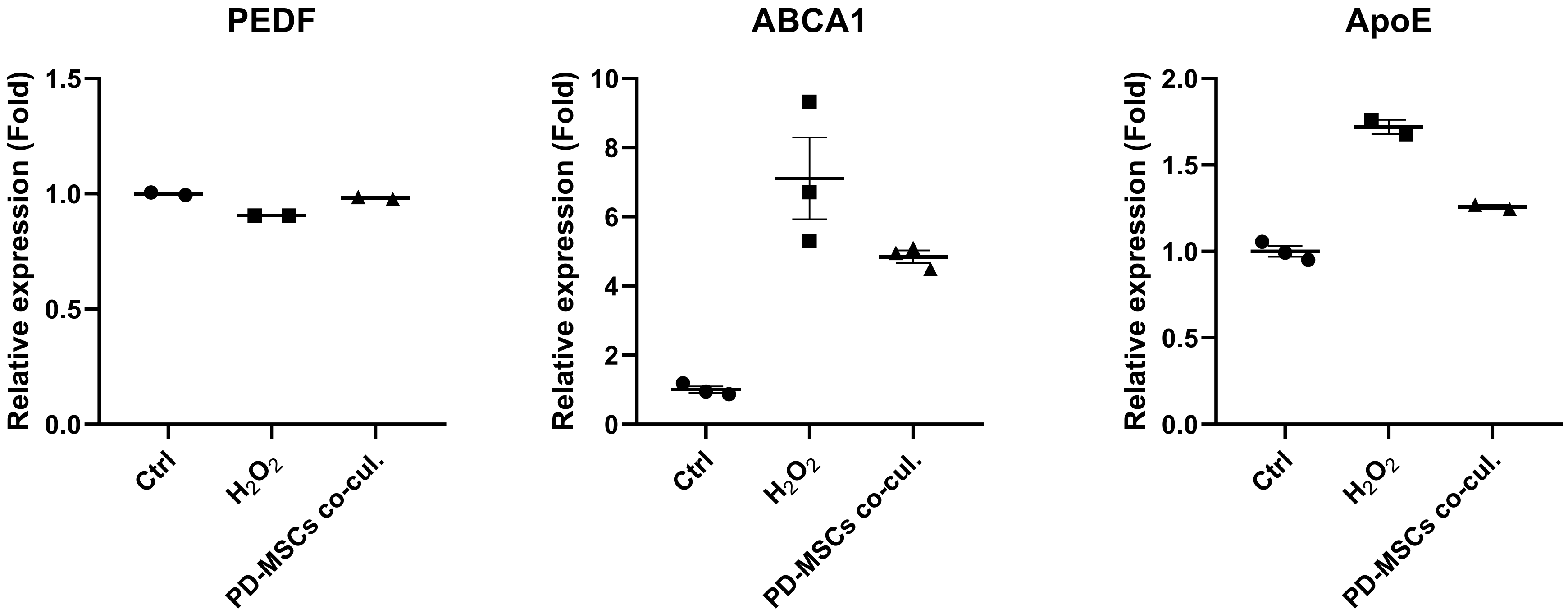
PEDF
ABCA1
ApoE
Relative expression (Fold)
1.5
1.0
0.5
0.0
10
8
6
4
2
0
2.0
Ctrl
H_2O_2
PD-MSCs co-cul.

Figure S3.

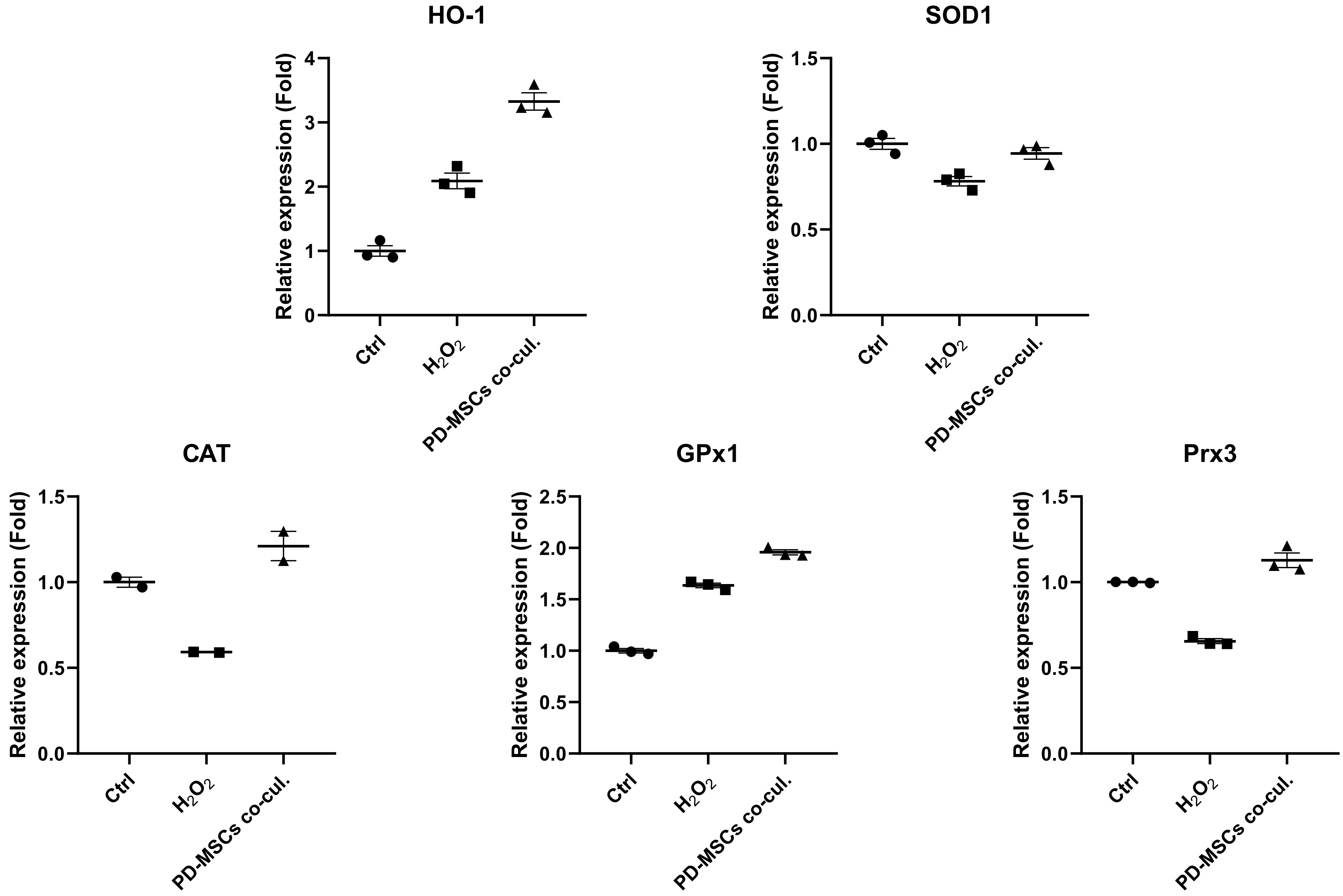

HO-1
SOD1
CAT
GPx1
Prx3
Relative expression (Fold)
Ctrl
H2O2
PD-MSCs co-cul.
0
1
2
3
4
0.0
0.5
1.0
1.5
2.0
2.5

Figure S4.

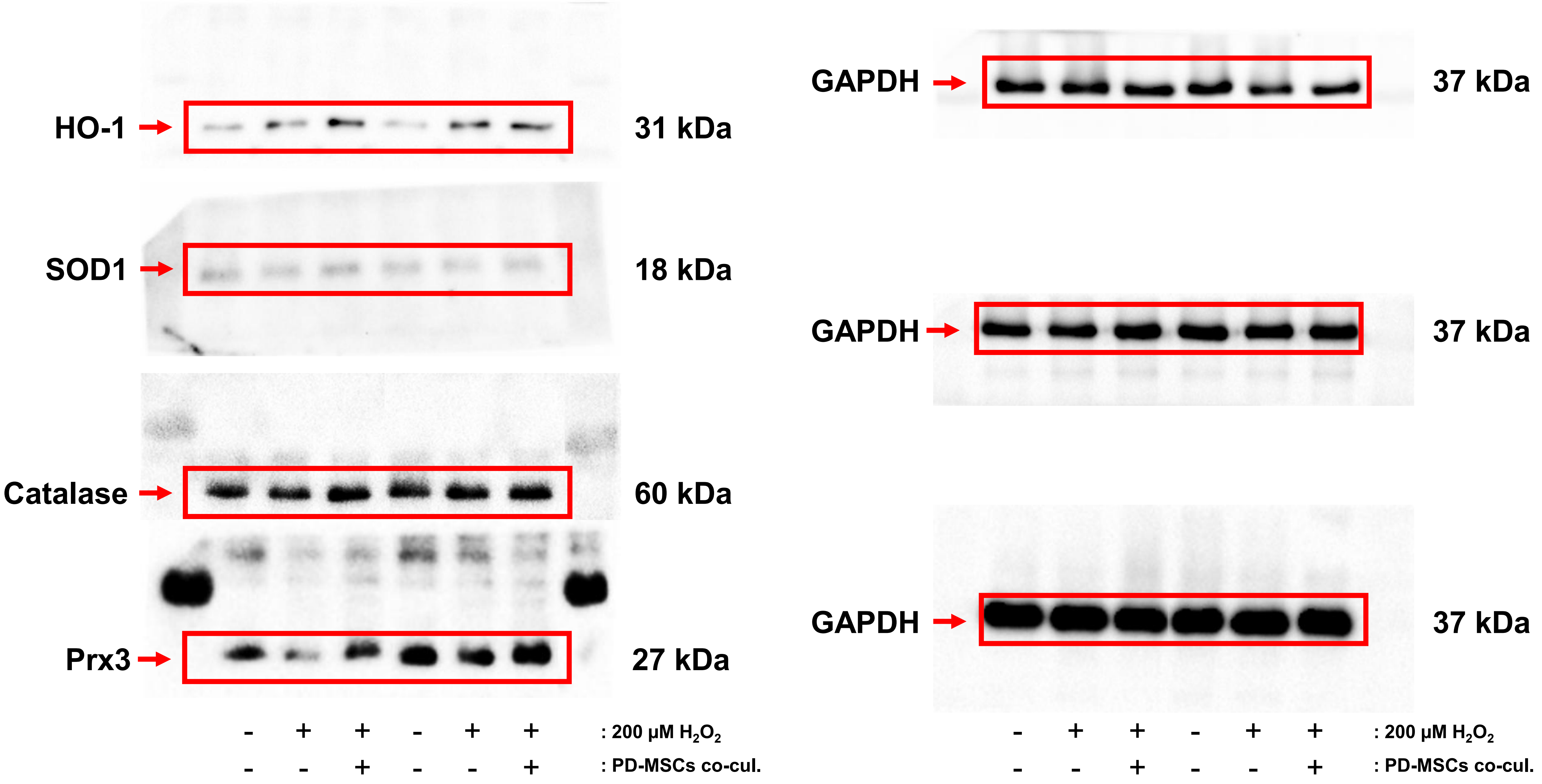
HO-1
31 kDa
SOD1
18 kDa
Catalase
60 kDa
Prx3
27 kDa
GAPDH
37 kDa
GAPDH
37 kDa
GAPDH
37 kDa
- + + - + + : 200 μM H_2O_2
- - + - - + : PD-MSCs co-cul.
- + + - + + : 200 μM H_2O_2
- - + - - + : PD-MSCs co-cul.

Figure S5.

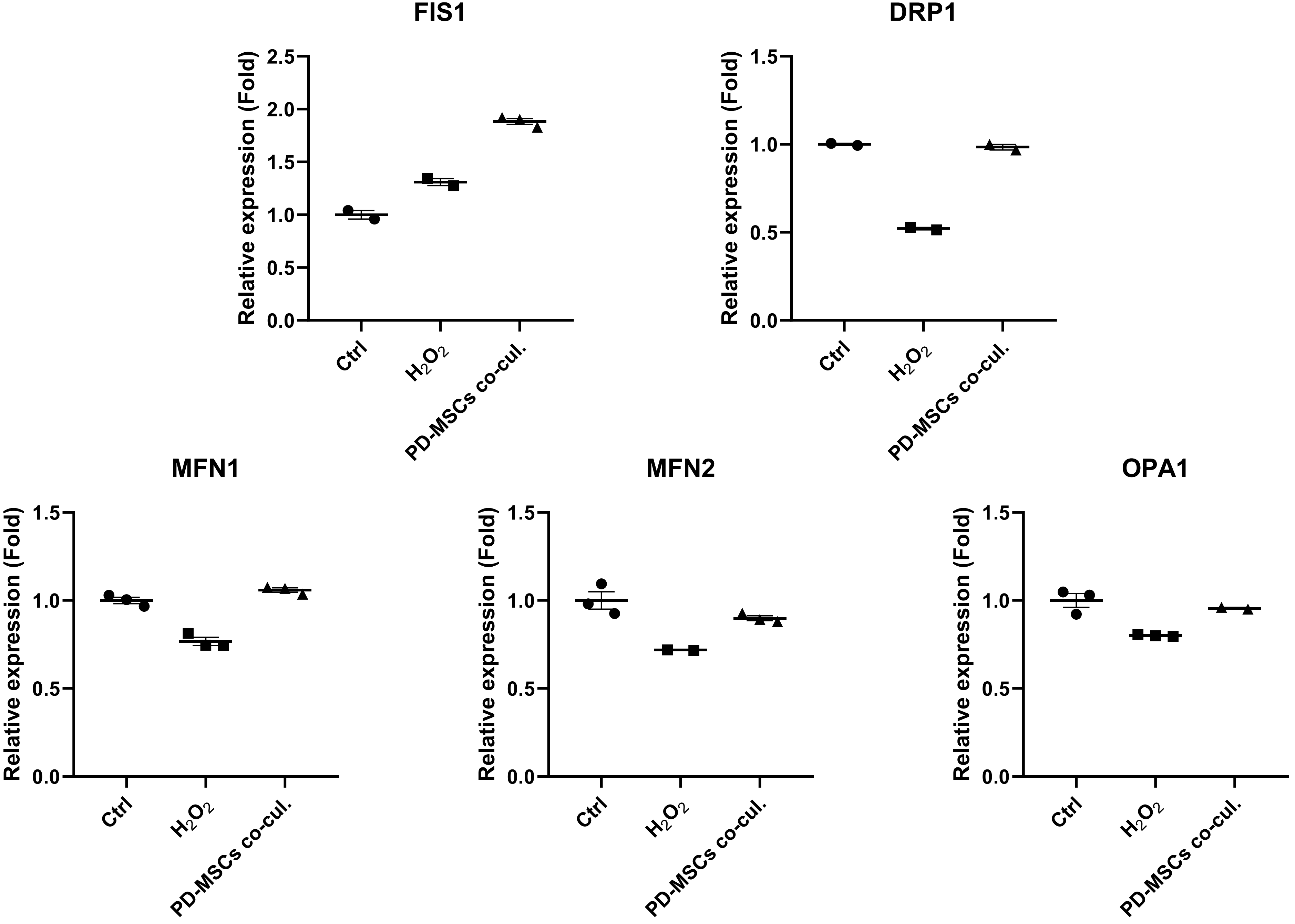
FIS1
DRP1
MFN1
MFN2
OPA1
Relative expression (Fold)
0.0
0.5
1.0
1.5
2.0
2.5
Ctrl
H_2O_2
PD-MSCs co-cul.

Figure S6.

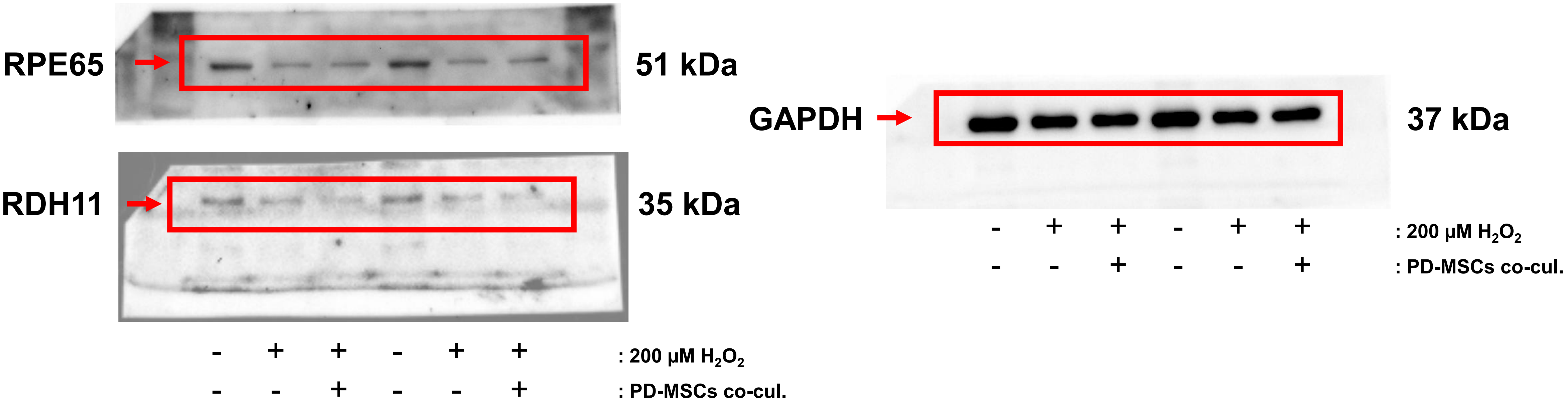

RPE65
51 kDa
RDH11
35 kDa
- + + - + +
: 200 μM H2O2
- - + - - +
: PD-MSCs co-cul.
GAPDH
37 kDa
- + + - + +
: 200 μM H2O2
- - + - - +
: PD-MSCs co-cul.

Figure S7.

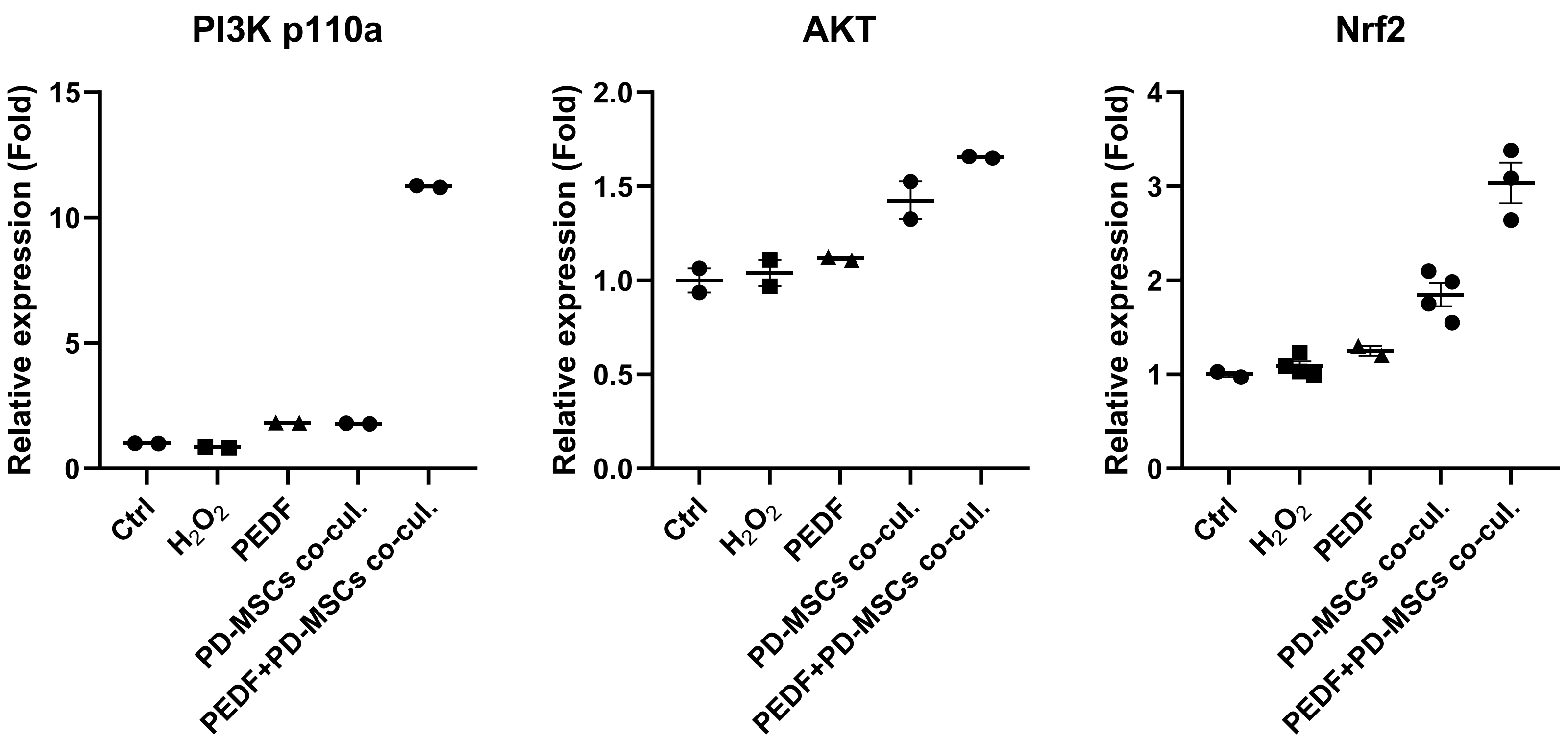
PI3K p110a
Relative expression (Fold)
0
5
10
15
Ctrl
H2O2
PEDF
PD-MSCs co-cul.
PEDF+PD-MSCs co-cul.
AKT
Relative expression (Fold)
0.0
0.5
1.0
1.5
2.0
Ctrl
H2O2
PEDF
PD-MSCs co-cul.
PEDF+PD-MSCs co-cul.
Nrf2
Relative expression (Fold)
0
1
2
3
4
Ctrl
H2O2
PEDF
PD-MSCs co-cul.
PEDF+PD-MSCs co-cul.

Figure S8.

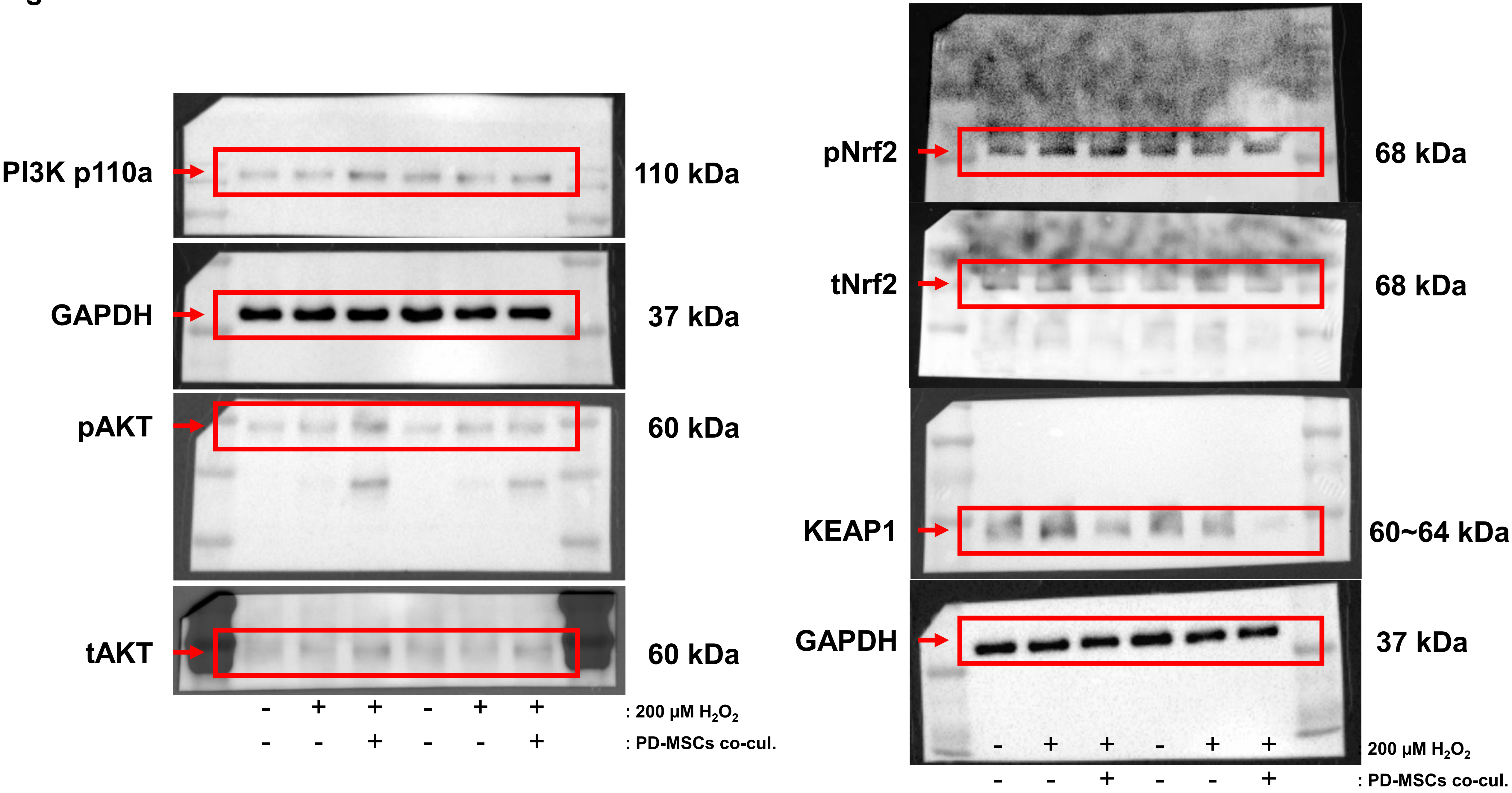
PI3K p110a
110 kDa
GAPDH
37 kDa
pAKT
60 kDa
tAKT
60 kDa
- + + - + +
: 200 μM H2O2
- - + - - +
: PD-MSCs co-cul.
pNrf2
68 kDa
tNrf2
68 kDa
KEAP1
60~64 kDa
GAPDH
37 kDa
- + + - + +
200 μM H2O2
- - + - - +
: PD-MSCs co-cul.
